# Fecal microbiota transplantation attenuates *Escherichia coli* infected outgrowth by modulating the intestinal microbiome

**DOI:** 10.1186/s12934-023-02027-z

**Published:** 2023-02-17

**Authors:** Yaping Wang, Yuanyuan He, Ying Liang, Han Liu, Xiushuang Chen, Muhammad Fakhar-e-Alam Kulyar, Asim Shahzad, Kunhua Wei, Kun Li

**Affiliations:** 1 Institute of Traditional Chinese Veterinary Medicine, College of Veterinary Medicine, Nanjing Agricultural University, Nanjing, 210095 People’s Republic of China; 2 Guangxi Key Laboratory of Medicinal Resources Protection and Genetic Improvement, Guangxi Engineering Research Center of TCM Resource Intelligent Creation, Guangxi Botanical Garden of Medicinal Plants, Nanning, 530023 China; 3 College of Veterinary Medicine, Huazhong Agricultural University, Wuhan, 430070 People’s Republic of China; 4 MOE Joint International Research Laboratory of Animal Health and Food Safety, College of Veterinary Medicine, Nanjing Agricultural University, Nanjing, 210095 People’s Republic of China; 5 Faculty of Veterinary and Animal Sciences, The Islamia University of Bahawalpur, Bahawalpur, 63100 Pakistan

**Keywords:** Fecal microbiota transplantation, Intestinal microbiome, *Escherichia coli*, Intestinal injury

## Abstract

**Background:**

Given the crucial role of gut microbiota in animal and human health, studies on modulating the intestinal microbiome for therapeutic purposes have grasped a significant attention, of which the role of fecal microbiota transplantation (FMT) has been emphasized.

**Methods:**

In the current study, we evaluated the effect of FMT on gut functions in *Escherichia coli* (*E. coli*) infection by using mice model. Moreover, we also investigated the subsequently dependent variables of infection, i.e., body weight, mortality, intestinal histopathology, and the expression changes in tight junction proteins (TJPs).

**Results:**

The FMT effectively decreased weight loss and mortality to a certain extent with the restoration of intestinal villi that resulted in high histological scores for jejunum tissue damage (*p* < 0.05). The effect of FMT on alleviating the reduction of intestinal TJPs was also proved by immunohistochemistry analysis and mRNA expression levels. Moreover, the abundance of health-threatening bacteria, belonging to phylum Proteobacteria, family Enterobacteriaceae and Tannerellaceae, genus *Escherichia-Shigella*, *Sphingomonas*, *Collinsella,* etc., were significantly increased, whereas beneficial bacteria, belonging to phylum Firmicutes, family Lactobacillaceae, genus *Lactobacillus* were decreased in the gut of infected mice. Furthermore, we sought to investigate the association of clinical symptoms with FMT treatment with modulation in gut microbiota. According to beta diversity, the microbial community of gut microbiota results reflected the similarities between non-infected and FMT groups. The improvement of the intestinal microbiota in FMT group was characterized by the significant high level of beneficial microorganisms with the synergistic decrease of *Escherichia-Shigella*, *Acinetobacter,* and other taxa.

**Conclusion:**

The findings suggest a beneficial host-microbiome correlation following fecal microbiota transplanatation for controlling gut infections and pathogens-associated diseases.

**Supplementary Information:**

The online version contains supplementary material available at 10.1186/s12934-023-02027-z.

## Introduction

In recent years, studies on the importance of intestinal microbiome in animal health have attained much attention. So, the understanding of healthy gut microbiota in a stable state has extended far beyond its role in digestion and energy acquisition due to its positive contribution to immune system development, behavior and host physiology [1–3]. The disruption of the gut microbiota homeostasis has been implicated in the pathogenesis of gastrointestinal disorders, e.g., IBD, metabolic diseases, immune diseases, and even mental diseases [4–6]. Among these disruption processes, the intestinal epithelial barrier plays essential physiological functions in host health. It’s composed of a tight junction, adhesion junction, gap junction, and desmosome that form multiple functional complexes that further prevent pathogens invasion [7, 8]. According to different researchers, the gut microbiome modulates intestinal barrier function by regulating tight junction proteins expression and intestinal capabilities [9, 10]. Therefore, such microbiota-targeted therapeutics might develop new strategies to cure the pathogen’s infection or its associated intestinal diseases.

Fecal microbiota transplantation (FMT) is used as therapy for some diseases associated with gut microbiota dysbiosis. Some studeis have already demonstrated the efficacy and safety of the FMT therapy [11–13]. Moreover, around 300 studies evaluated FMT for various primarily gastrointestinal conditions, such as inflammatory bowel disease [14]. The diversity of the intestinal microbiome is more abundant in FMT, which is important for defense against enteric pathogens. Further, it  improves gut microbiota dysbiosis [15]. In children with autism spectrum disorder, Kang et al. [16] reported that symptoms of constipation, indigestion, diarrhea, and abdominal pain had been improved following FMT. In another study, Zhao et al. [17] manifested that FMT might be helpful for Parkinson’s disease (PD) treatment. Similarly, Zhang et al. [18] proved that FMT is useful in curing Colitis in mice by gut microbiota regulation. Moreover, over the past decade, FMT has attained various attention due to its effectiveness as a therapy for treating *Clostridioides difficile* infection (CDI). Although it appears to be effective in CDI, but its capabilities in other enteric pathogen infections, e.g., *E.coli*, remain poorly understood. Moreover, it is unclear whether it plays a beneficial role through changes in host physiological processes driven by alteration of intestinal microbial composition and structure. The etiologic agents in infections carries virulence factors that could cause bacteremia and sepsis through intestinal and extraintestinal [19]; of among, *E.coli* is a frequent bacterial pathogen [20]. The increased use of antibiotics in treating bacterial pathogen infection has led to an epidemic of antibiotic resistance bacteria, posing severe challenges to the therapeutic effect of patients infected with resistant pathogens. World Health Organization (WHO) announced a worldwide health security emergency because antibacterial medicine is losing its effectiveness. Therefore, identifying different ways that have therapeutic effects on microbial infectious diseases is getting crucial.

Based on effectiveness in CDI, we hypothesized that FMT regulates intestinal epithelial barrier injury caused by *E. coli* by modulating the composition and structure of intestinal microbiota. Therefore, the purpose of current study was to investigate the interplay between the intestinal microbiome and host following FMT from high-throughput sequencing, intestinal histopathology, and immunohistochemistry by establishing an *E. coli*-infected mice model. The current study provided a theoretical underpinning for using FMT as a viable treatment for gut microbial regulators.

## Materials and methods

### Animals

**Fig. 1:**
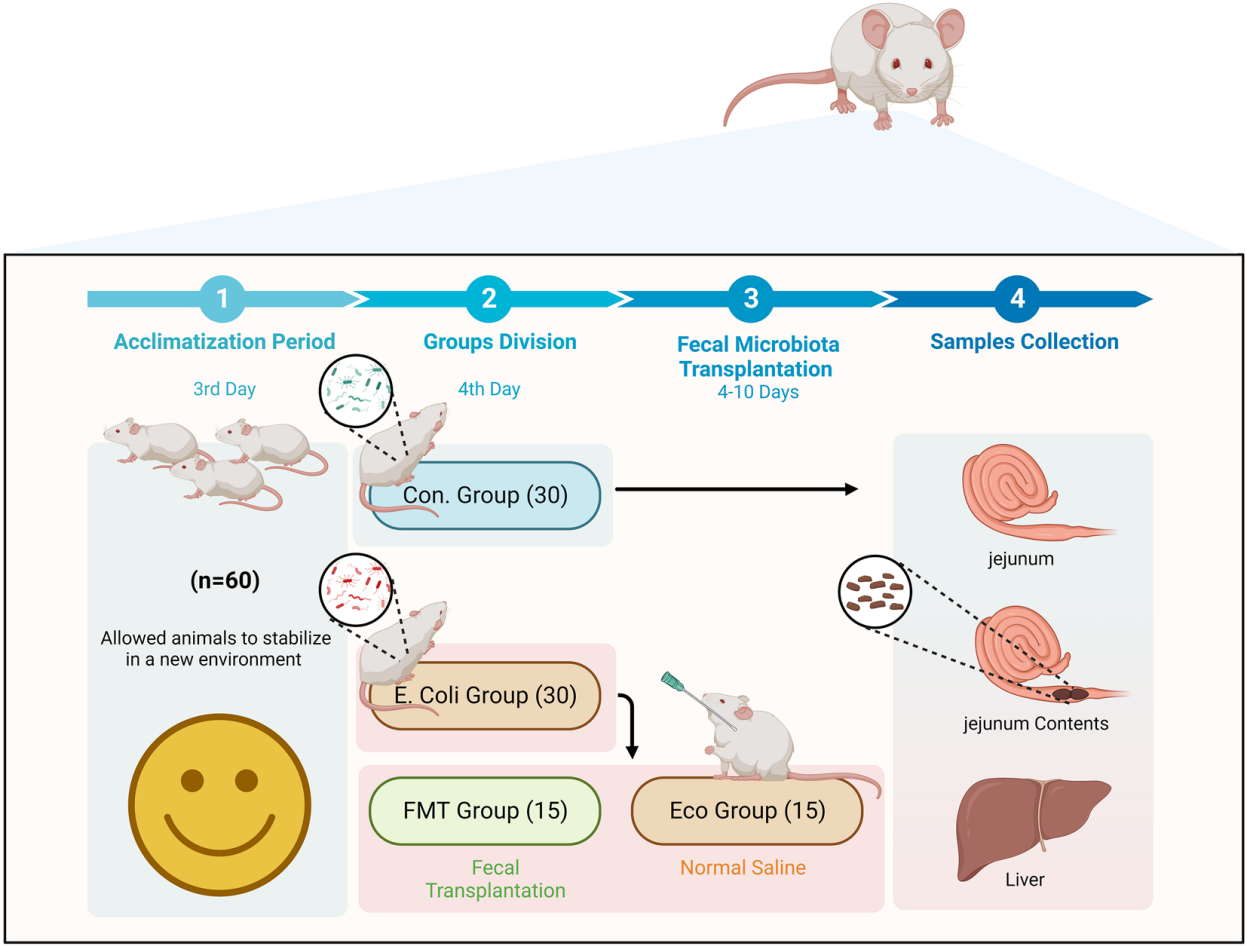
The flowchart of experiment

Six-week old BALB/c specific-pathogen-free mice (a total of 60 mice, half males and half females) were purchased from Jiangsu Experimental Animal Center (Jiangsu, China). All animals were placed under standard conditions (12 h light/dark cycle; temperature: 23 ℃ ± 1 ℃; relative humidity: 50% ± 5%) for 3 days before performing any experiment procedures. The mice obtained a standard chow diet and water during the trial period. Mice were randomly divided into the control group (named Con group, n = 30, not inoculated with *E.coli*) and *E.coli* group (n = 30, inoculated orally with *E.coli* suspension 1 × 10^9^ CFU for 3 days). The pathogenic *E.coli* strain (Enteroinvasive *Escherichia coli* BNCC340975) was obtained from the College of Veterinary Medicine, Huazhong Agricultural University, referring to our previous research [21]. The infected mice (*E.coli* group) were inoculated orally with FMT (named FMT group, n = 15) and normal saline (named Eco group) for a week (from day 4 to day 10). The control mice were assigned as the source of donor stool. The initial and final body weights of all mice were recorded. Then mice were sacrificed via cervical dislocation on day 13 for collecting liver and jejunum tissues and contents aseptically. All samples were kept at − 80 ℃ until further analysis. Moreover, a timeline flowchart of the current experiment is shown in Fig. 1.

### Fecal microbiota transplantation

100 mg of fresh fecal pellets were collected daily from donor mice immediately after defecation, suspended in 1 mL normal saline, vortexed and centrifuged at 2500 × g for 1 min each. Then 100 μL aliquots of supernatant from the stool samples were administered to recipient mice by gavage immediately to minimize the alterations in the fecal microbiome composition [22].

### Analysis of intestinal injury and histopathology

Tissues from mice in all groups were collected and rinsed gently with PBS. All specimens were fixed in 4% paraformaldehyde for 48 h, cut into 4 μm thick sections, followed by dehydration, embedding, and hematoxylin and eosin (H&E) staining. The light microscopic was used to examine tissue injury by a pathologist who was blind to the treatment according to the method described in a previous study [23].

### Immunohistochemistry analysis

Paraffin sections of embedded jejunum tissues were sectioned into 4 mm thick slices. The sections were dewaxed in xylene and rehydrated with aqueous alcohol solutions; then, they were incubated in 10% H_2_O_2_ for 10 min to remove endogenous enzymes. The sections were then incubated at 4 °C overnight with primary antibodies against ZO-1, Occludin, and Claudin-1 (purchased from Proteintech Group, Inc, USA). After washing with TBS (n = 3), the sections were reacted with biotinylated HRP labeled rabbit anti-goat secondary antibodies for 30 min. Subsequently, the sections were stained with diaminobenzidine (DAB) using a commerical kit (ABclonal technology, China) and finally counterstained with hematoxylin. After that, the expression and localization of ZO-1, Occludin, and Claudin-1 were examined using OLYMPUS microscope analyzer.

### Extraction of jejunum tissue mRNA and determination of expression levels

Total RNA from jejunum was extracted using Trizol reagent (Invitrogen) and converted to cDNA using the Reverse Transcription Kit (Takara, Japan). Next, the quality and the concentration were examined using NanoDrop nucleic acid analyzer (ThermoFisher Scientific, USA). The mRNA expression levels of the ZO-1, Occludin, and Claudin-1 were detected using real-time quantitative polymerase chain reaction (PCR). The primers of target genes used in this research are shown in Additional file 1: Table S1. The mRNA expression level results were calculated using the 2^–△△Ct^ method.

### Microbial genomic DNA extraction

Genomic DNA in intestinal contents was extracted using QIAamp DNA Mini Kit (QIAGEN, Germany) following the manufacturer's instructions, with the addition of an incubation step before bead-beating. The DNA concentration was quantified using NanoDrop spectrophotometer (Thermo Scientific, Wilmington), while the DNA integrity was evaluated by 1% gel electrophoresis (voltage: 150 V; electrophoresis time: 40 min). Then Zymo Onestep Inhibitor Removal kit (Zymo Research PN. D6035) was used to inhibit downstream enzymatic reactions. Finally, DNA extracts were kept at − 80 °C for further processing.

### 16S rRNA genes amplification and high-throughput sequencing

The V3–V4 hypervariable regions of the bacterial 16S gene were amplified along with the following primers: 308 Forward Primer: 5′-ACTCCTACGGGAGGCAGCA-3′ and 806 Reverse Primer: 5′-GGACTACHVGGGTWTCTAAT-3′. The PCR cycling was performed at 95 °C for 3 min (initial melt), followed by 30 cycles of amplification with a 95 °C for 30 s, 55 °C for 1 min (annealing step), and a 72 °C extension step for 1 min. A final, 10 min extension step was performed following the last cycle.

The PCR amplicons were indexed, cleaned, and normalized before sequencing on Illumina platform, resulting in paired-end reads. Then PCR products were purified and assessed using AMPure XP beads (AGENCOURT) and 1% gel electrophoresis. A sequence library was generated, using the purified PCR amplification products for single peak libraries, having 2 nM concentration. Then high-throughput sequencing was performed using the paired-end method on Illumina HiSeq platform (Illumina, San Diego, USA).

### Bioinformatics and statistical analysis

The raw sequence reads were processed by DADA2 workflow implemented in the R package (v1.12.1) to identify amplicon sequence variants (ASVs), trim adapter data, and chimeras were deleted. Before bioinformatics analysis, raw sequences from high-throughput sequencing were performed to obtain more reliable, and high-quality sequencing reads (effective reads) by the following procedures: (1) those identical reads potentially generated by PCR amplification were removed; (2) Trimmomatic v0.33 software was used to filter those reads to delete adapters using, and the sequences that too short (N < 60 bp) and low-quality (< 15 M reads after QC) were discarded by using cutadapt 1.9.1 software; (3) those sequences contaminated by adapter were deleted (maximal 3 bases mismatch allowed); (4) sequences with low complexity were deleted (reads with consecutive same more than 10 bp). Subsequently, Operational taxonomic unit (OTU) clustering was proceeded at over 97% sequence similarity using Usearch software. Mapping OTU to the SILVA database using Quantitative Insights Into Microbial Ecology platform (QIIME, v1.8.0) to assign taxonomy. All visualizations were performed in R software (v3.6.1) unless otherwise specified. The rarefaction curve, Shannon curve, and species accumulation curve, which reflect sequencing depth and quality, were analyzed by Mothur (v1.31.2) and R software. Venn diagram visually shows the number of unique and common OTUs among groups. Chao1 index, ACE index, Simpson index, and Shannon index, which reflect alpha-diversity, were calculated using QIIME software, while Good’s coverage index evaluated species coverage. The β diversity was assessed by Non-Metric Multidimensional Scaling (NMDS), unweighted pair-group method with arithmetic mean (UPGMA), and clustering heatmap of samples. Linear discriminant analysis effect size (LEfSe) could detect the statistical difference in the abundance of microorganisms among groups. Based on Metastats analysis, normalized OTU was used to calculate the relative abundances of taxa classification (reads summarized at the phylum and genus levels) in all samples. The QIIME software built-in scripts were used to construct the genus-level phylogenetic tree and were visualized by R software at last. Spearman correlation analysis was performed to find data with a correlation greater than 0.1 and p < 0.05 to construct a correlation network and then was visualized using python software. P-values of < 0.05 were considered significant differences, the data was recorded as means ± SD.

## Results

### Administration of E.coli induced weight loss and mortality in mice

**Fig. 2:**
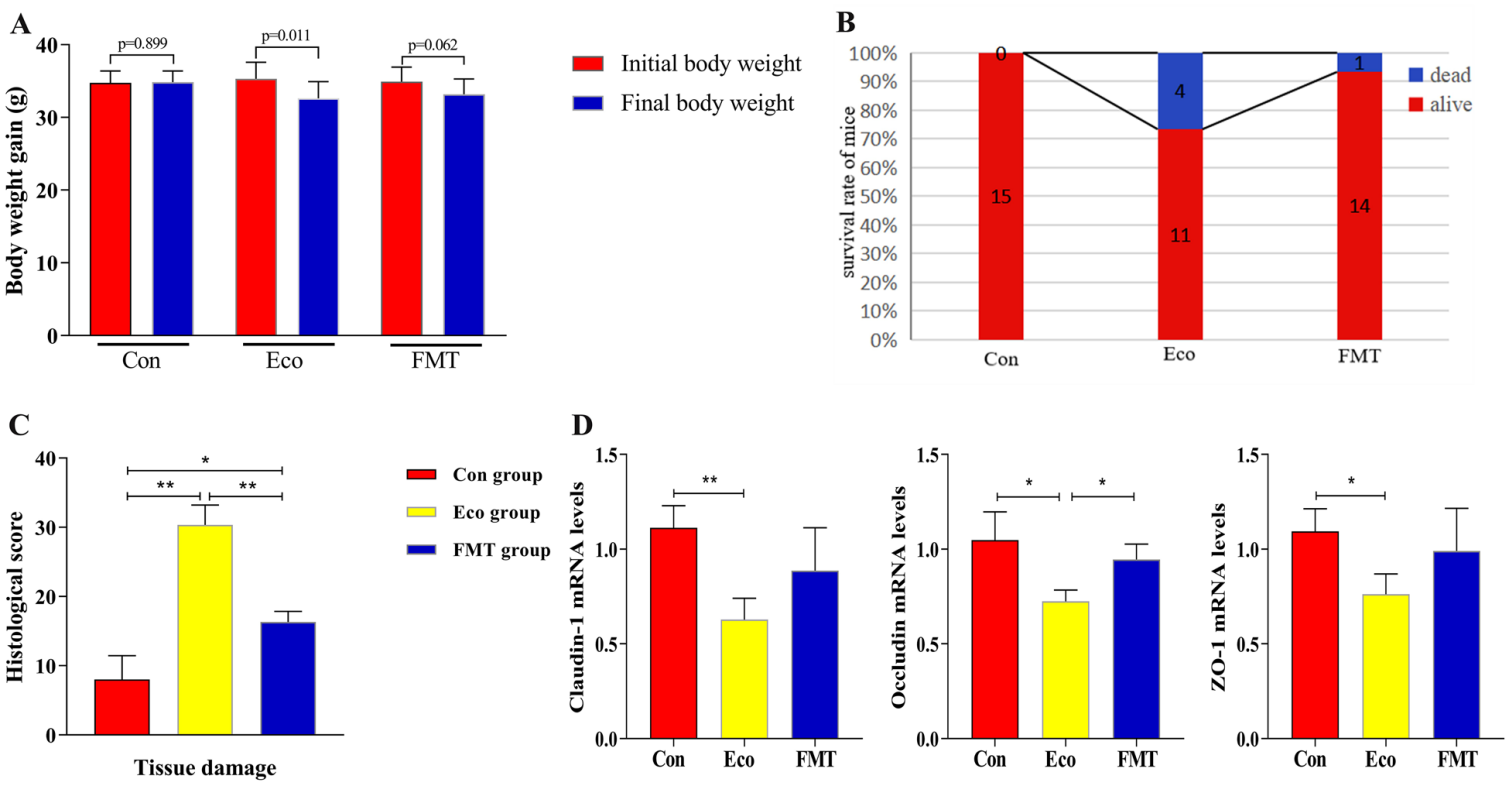
Evaluation of body weight loss, survival rate and gut damage after *E. coli* infection. **A**, body weight. **B**, survival rate. **C**, histological score. **D**, the mRNA expression levels of TJPs in the jejunum. * *p* < 0.05, ** *p* < 0.01

The *E.coli* infection caused weight loss (*p* < 0.05) and increased mortality in mice of Eco group (26.7%) compared with Con group. However, the weight loss (*p* > 0.05) and mortality (7%) of the recipient mice were reduced following FMT (Fig. 2A, B). Moreever, current data showed that FMT decreases mortality and weight loss in *E.coli* infected mice model.

### The histopathology effects of FMT on liver and jejunum

**Fig. 3:**
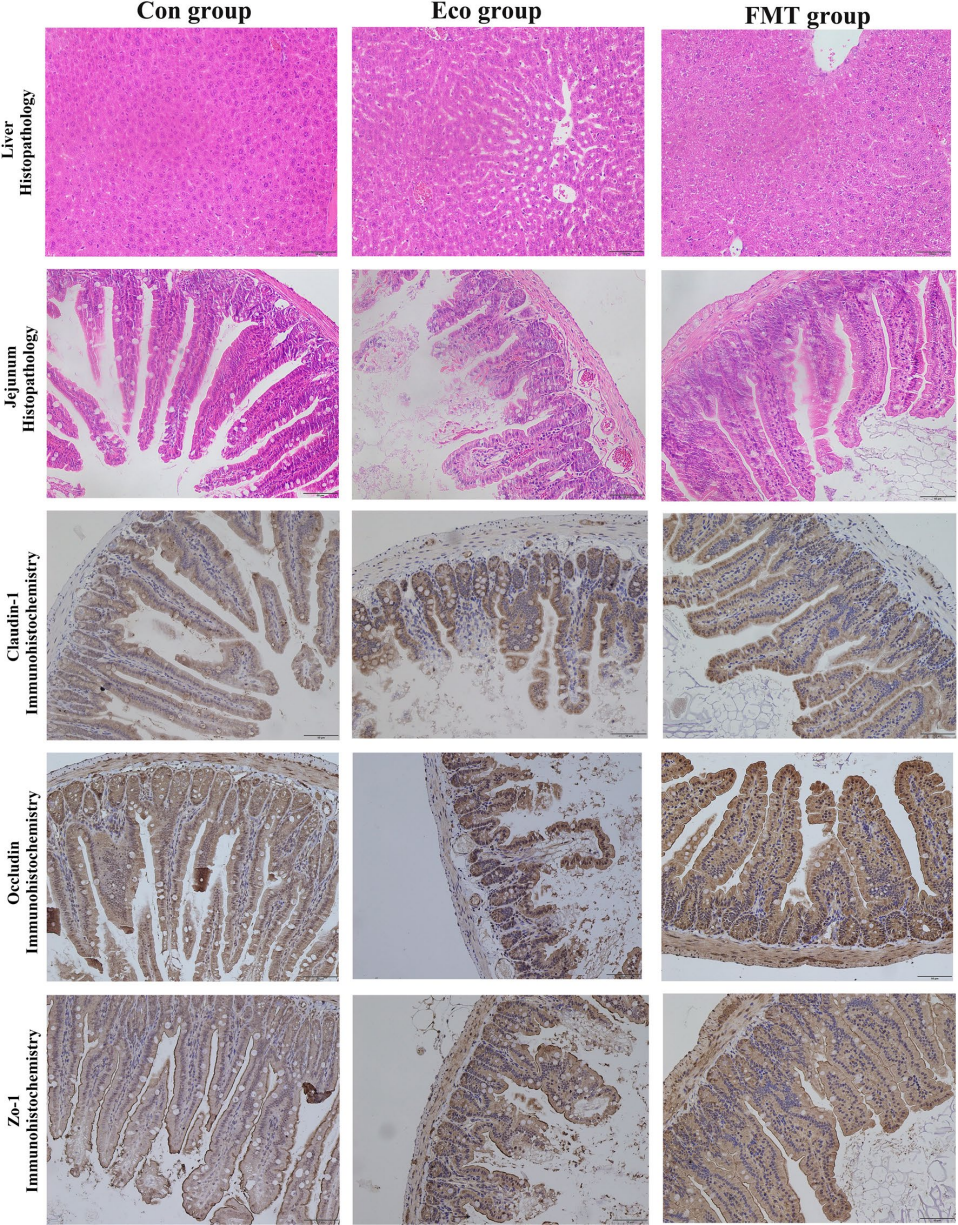
Jejunum histopathological and immunohistochemical changes in different groups. Bar = 50 µm

Pathogens cause more serious systemic infections by damaging the digestive tract, so we further examined whether FMT relieved the damaged structure of the digestive system driven by infection in mice. Consistent with body weight loss, the intestinal villi of infected mice (Eco group) were arranged irregularly and decreased in length. The structure of intestinal lamina propria was damaged and even fragmented, resulting in the highest histopathological scores for tissue damage (Fig. 2C, Fig. 3). In addition, the length of intestinal villi in FMT group was similar to the control group, the arrangement was evenly and orderly, and intestinal walls were intact. Moreover, the liver of infected mice was dark red with blunt edges, and congestion; while 7 days after FMT treatment, it was found that the liver congestion was significantly relieved (Fig. 3).

### The immunohistochemistry analysis of gut tight junction proteins

Gut barrier is a first line of defence to prevent enteric pathogen invasion. The barrier has epithelial tight junction proteins (TJPs) that contribute to an efficient gut barrier. Therefore, we examined the effects of FMT on the regulation of barrier function formation. We used antibodies against ZO-1, Occludin, and Claudin-1 to immunostain intestinal tissue sections. The positive particles were stained dark brown or yellow brown in antigenic sites. Compared with the control group, the numbers of ZO-1, Occludin, and Claudin-1 positive cells decreased significantly in infected mice (Fig. 3). In addition, the positive expression of ZO-1, Occludin, and Claudin-1 in FMT group was increased, compared to Eco group.

### Analysis of the mRNA expression levels

As shown in Fig. 2D, the mRNA expression of TJPs in jejunum tissues of Eco group decreased significantly compared to control group (*p* < 0.05 or *p* < 0.01). After FMT, the Occludin expression increased significantly (*p* < 0.05); however, the expression levels of the other two proteins increased gently with no significant difference. These data were consistent with the trend of immunohistochemical results.

### Analysis of 16S rDNA sequence data

**Fig. 4:**
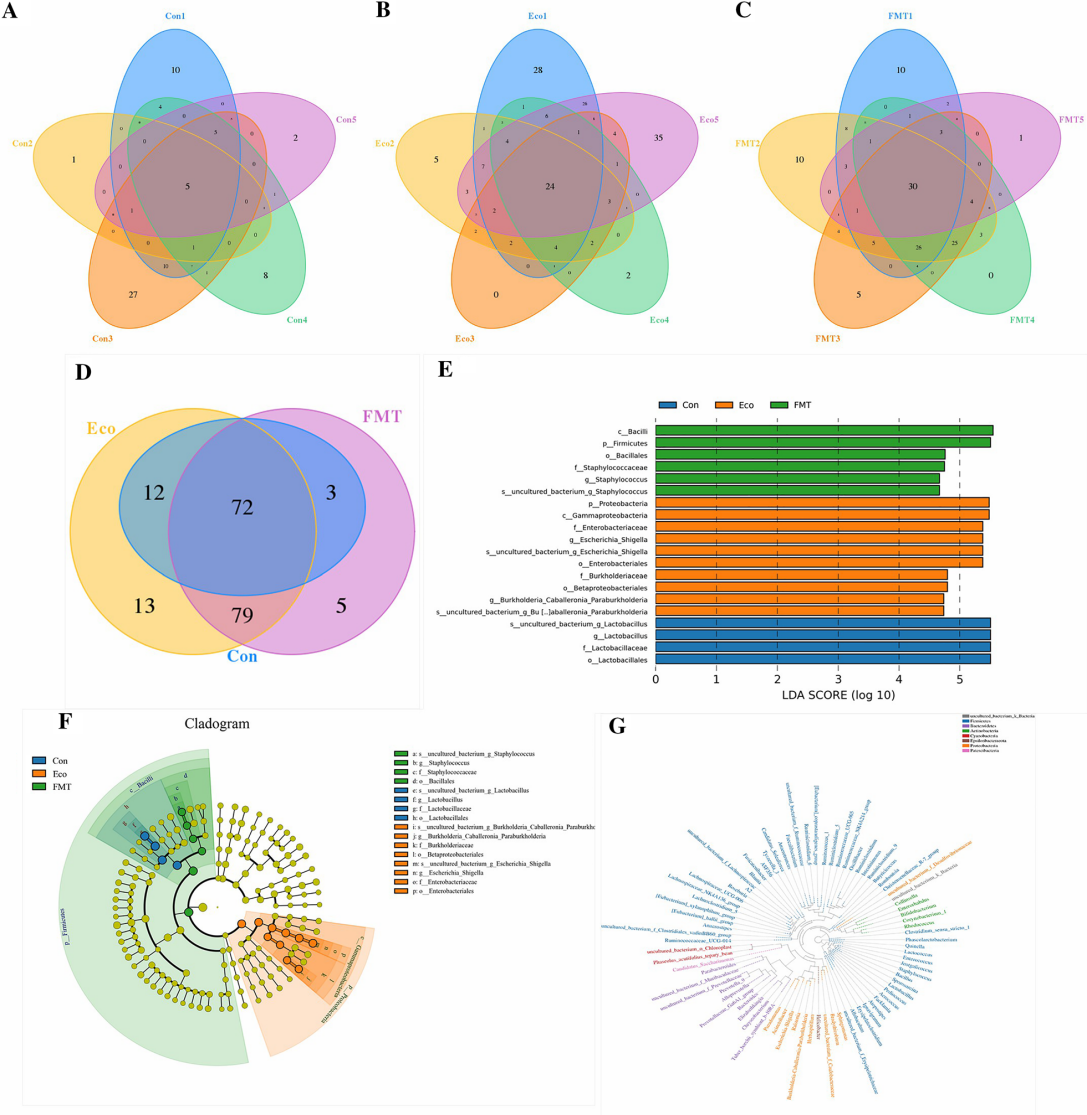
Structural changes of jejunum microbiota. **A**–**C** represent the number of OTUs in groups, i.e., Con, Eco, and FMT. **D** represent the number of common and core OTUs among the groups. **E**, **F** represent Histograms and Cladogram of enriched taxa based on LEfSe determinations, respectively. Microbial classification with an LDA score ≤ 5 was selected as biomarker microorganisms (*p* phylum, *c* class, *o* order, *f* family, *g* genus). **G** represented the genus-level phylogenetic tree

**Fig. 5:**
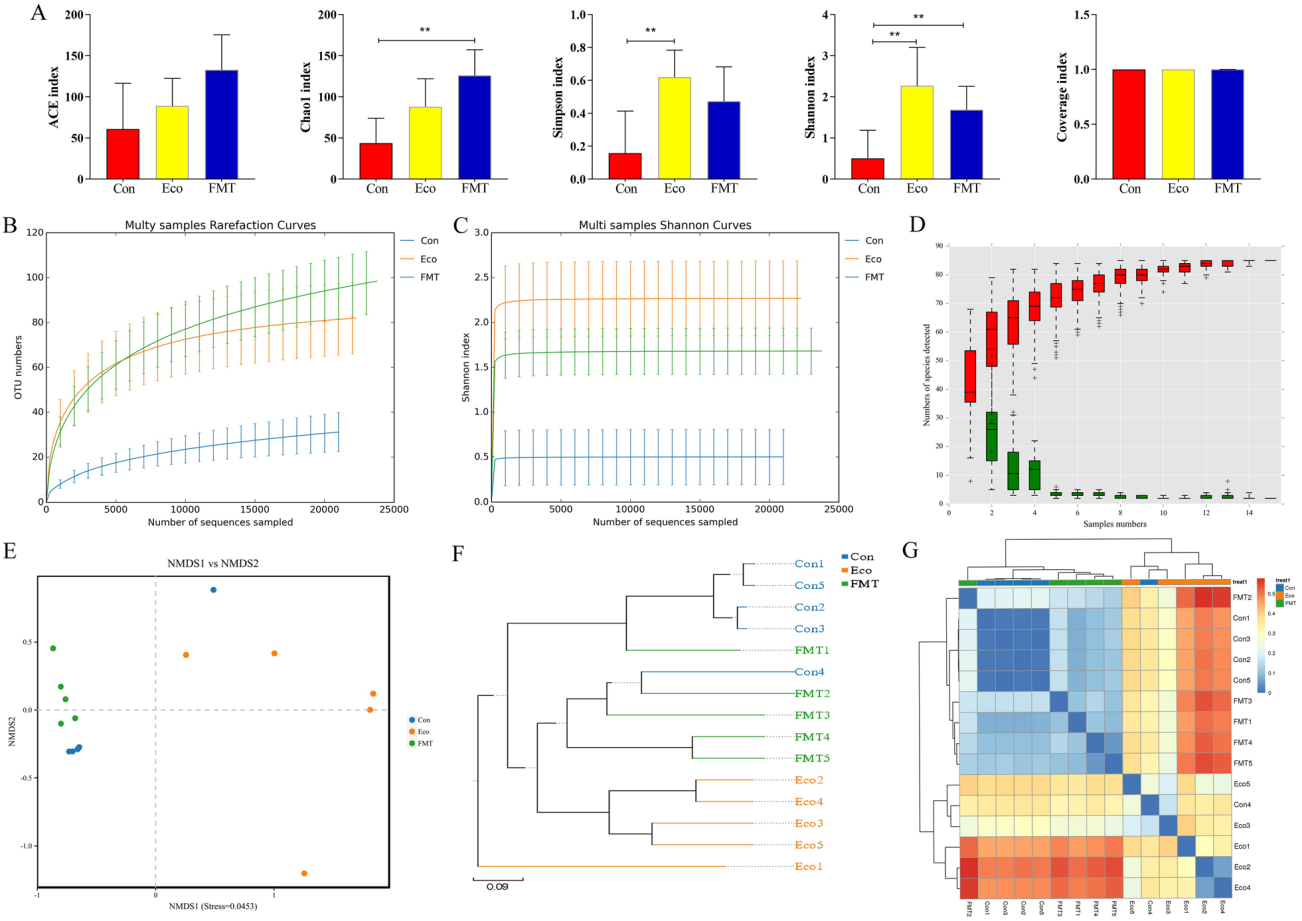
Analysis of sequencing quality, gut microbial alpha, and beta diversities. **A**, Alpha diversity; **B**, Rarefaction curve; **C**, Shannon curve; **D**, Species accumulation curve; **E**, NMDS, The closer samples on the coordinate map, shows higher similarity between samples; **F**, UPGMA, The closer samples are more similar in composition; **G**, Clustering heatmap, The color gradient from blue to red indicated the distance between samples from near to far. * *p* < 0.05, ** *p* < 0.01

The current high-throughput sequencing generated 696,824 raw sequences (Table 1). After optimizing the original data, effective reads based on 97% similarity were collected for the samples (393,300). The total OTUs assigned were 87, 176, and 159 for the Con, Eco, and FMT groups, respectively (Fig. 4A–C). Next, Venn diagram was drown-based amplicon sequence to reflect common and unique species among three groups. A total of 72 core OTUs were found from all samples, comprising approximately 17% of the total OTUs. Additionally, 13 unique OTUs were identified in Eco group, and 5 OTUs were uniquely found in the FMT group (Fig. 4D). Rarefaction Curve, Shannon index, and species accumulation curve trended to flat and the coverage of all samples over 99%, suggested that sample size, diversity of microorganisms, and depth of sequencing were sufficient and reliable to perform the final bioinformatics analyses (Fig. 5B–D).

**Table 1:** The information of 16S rDNA sequence data

Sample ID	Raw reads	Clean reads	Effective reads	AvgLen(bp)
Con1	46,418	30,186	28,675	429
Con2	37,061	23,856	22,656	429
Con3	36,515	24,197	23,186	429
Con4	38,927	21,723	21,106	428
Con5	43,637	27,512	26,394	429
Eco1	52,340	26,968	25,652	413
Eco2	53,591	28,887	27,575	428
Eco3	42,181	25,921	23,725	429
Eco4	50,205	28,518	27,275	429
Eco5	42,357	24,119	22,994	427
FMT1	46,772	29,576	25,889	428
FMT2	47,014	28,979	26,606	427
FMT3	48,005	30,740	26,168	429
FMT4	48,568	30,357	28,632	429
FMT5	63,233	41,376	36,767	429

### Alpha‑and beta-diversity of microbial communities

We next evaluated the effect of FMT on the intestinal microbiome of infected mice. We performed multiple analysis methods to assess the alpha diversity of the intestinal community more comprehensively, i.e., ACE and Chao1 (species directly reflect the richness of microbial community), Shannon and Simpson (directly reflect the species diversity of microbial community) indexes (Fig. 5A). Following the Chao1 ecological parameters, 60.96, 88.90, and 132.74 average indexes were found in samples at groups Con, Eco, and FMT, respectively. Whereas the ACE index showed 43.80, 87.96, 125.96, which suggested that the FMT group had relatively higher community richness. Inter-group analysis of the Simpson index intuitively showed that the bacterial diversity in the Eco group was significantly higher than Con group (*p* < 0.01). Consistently, a lower Shannon index was found in the Con group than Eco group (*p* < 0.01). Generally, the results showed that Con had relatively lower bacterial richness and diversity.

To further dissect the difference and similarities of gut bacterial communities among the three groups, we calculated β-diversity by using distance method, i.e., NMDS, UPGMA, and Cluster heatmap analysis in order to evaluate the diversity between individuals or groups. In the scatter plot from NMDS, samples in Eco group were decentralized distribution with lower similarity with samples in Con and FMT groups. We simultaneously obtained similar conclusions in the distance matrix of UPGMA and Cluster heatmap (Fig. 5E–G).

### Significant changes in the gut bacterial community structure following FMT

**Fig. 6:**
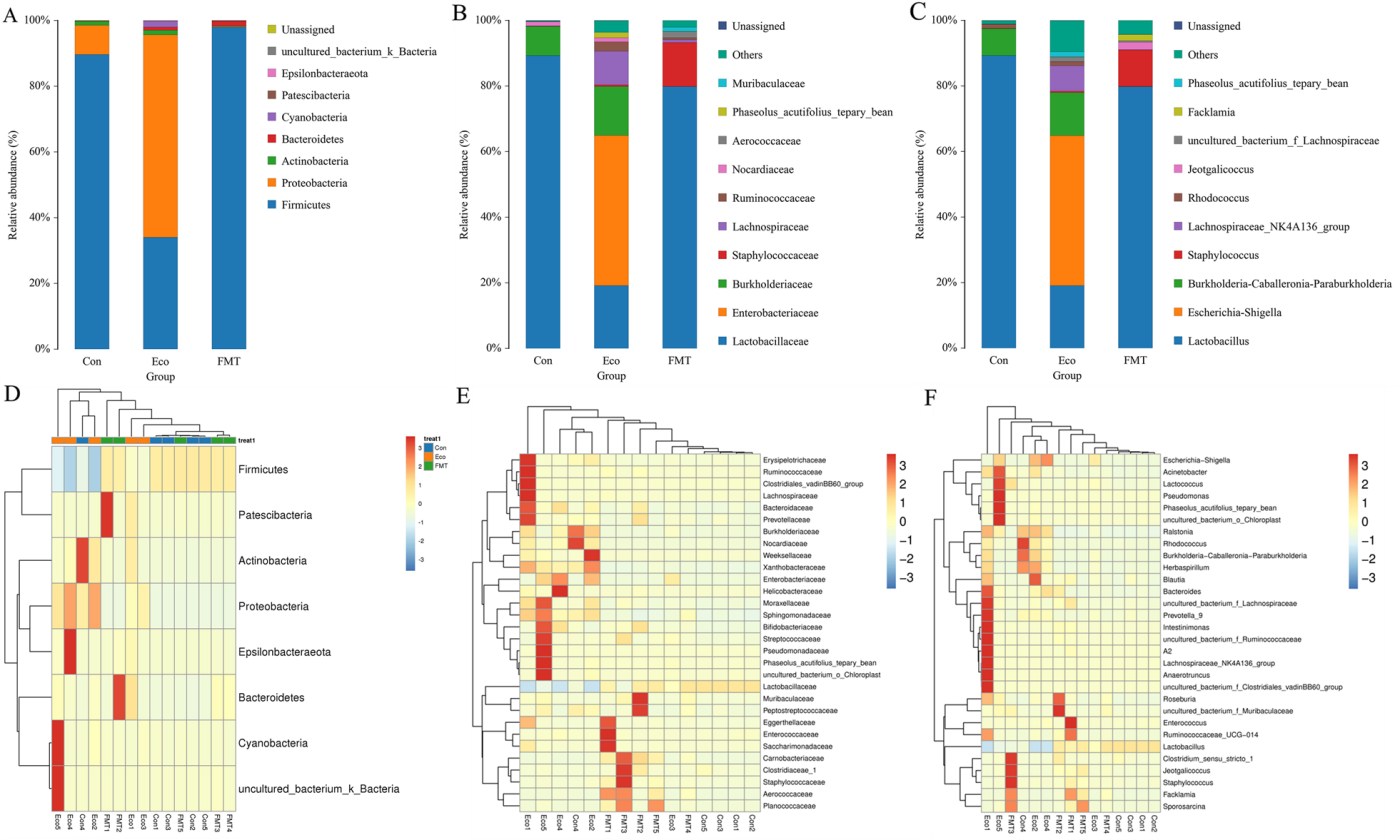
Relative abundances of jejunum microbiota at multiple levels. **A**–**C** The phylum, family, and genus level among groups. **D**–**F** The phylum, family, and genus levels between individual samples

The distribution and relative abundance of taxa in phylum, family, and genus levels samples were used to evaluate differences among microbial communities (Fig. 6). Importantly, the main dominated abundance of several intestinal microbiota that had been detected in FMT was comparable to Con, which was consistent with the findings of Beta diversity, regarding microbial communities between control and FMT groups. Gram-positive Firmicutes were the most predominant (over approximately 89.648% of the intestinal microbiome) in both Con and FMT groups, while phylum Proteobacteria maximum colonized in the intestinal tract of Eco group (average of 61.564%). Predictably, the family Enterobacteriaceae and Lachnospiraceae were the most abundant in Eco group (45.785%, 10.332%), whereas both of them were almost absent in the Con (0.002%, 0.008%) and FMT (0.082%, 0.851%) groups. Interestingly, the most abundant family in Con and FMT was Lactobacillaceae (89.345%, 79.841%), which was rarely colonized in the Eco group (19.166%). *Escherichia-Shigella* (Con vs Eco vs FMT: 0.002% vs 45.785% vs 0.008%) belonging to the family Enterobacteriaceae phylum Proteobacteria, while *Lactobacillus* (Con vs Eco vs FMT: 89.341% vs 19.120% vs 79.832%) belonging to family Lactobacillaceae phylum Firmicutes were found abundant among groups. Moreover, we found consistancy in abundance of groups regarding to phylum, family, and genus levels.

### Significant changes in the microbial taxonomic compositions following FMT

**Fig. 7:**
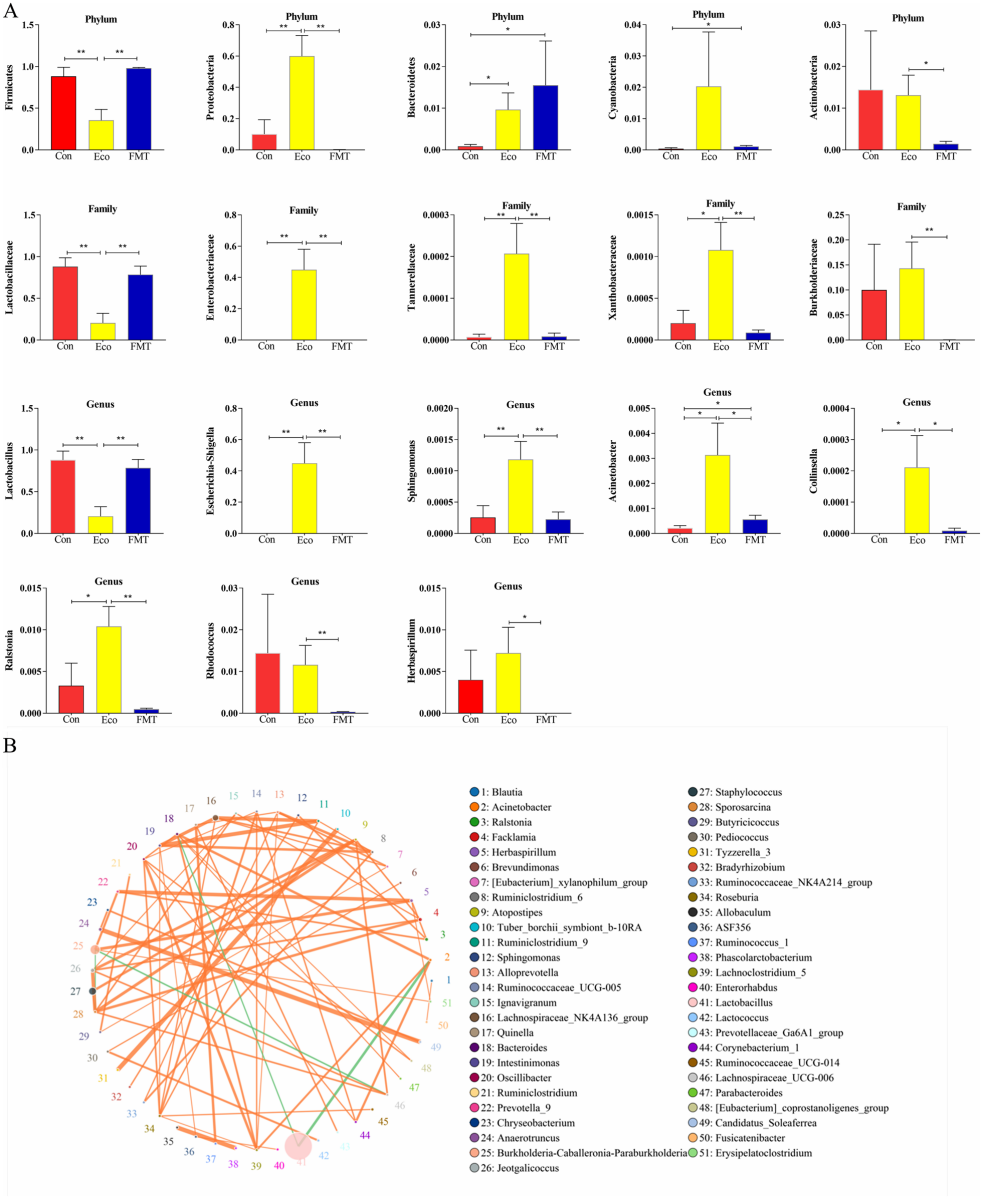
The correlation of microbial species and the significant changes in microbial composition between groups. **A**, Correlation network diagram based on spearman analysis; **B**, Significantly changes in microbial composition between groups. * *p* < 0.05, ** *p* < 0.01

To further investigate how the microbial taxonomic compositions at multi-levels altered on *E.coli* infected mice following FMT, Metastats analysis was performed to evaluate the differentially abundant phyla, family, and genera among groups (Fig. 7A). At the phylum level, the results demonstrated that Firmicutes (*p* < 0.01) were significantly more abundant both in Con and FMT groups than in the Eco group; however, the abundance of Proteobacteria (*p* < 0.01) and Bacteroidetes (*p* < 0.05) were significantly increased in the Eco group than in the Con group. Significantly, compared with Eco group, we found a decrease in the relative abundances of Proteobacteria (*p* < 0.01) and Actinobacteria (*p* < 0.05) following FMT. The differences in the relative abundance between the Eco group and the other two groups were consistent with the previous analysis; that was, the similarity of the sample was higher between Con and FMT groups. Lactobacillaceae (*p* < 0.01) and *Lactobacillus* (*p* < 0.01) were detected to be more abundant in both Con and FMT groups than in Eco group, whereas Enterobacteriaceae (*p* < 0.01) and *Escherichia-Shigella* (*p* < 0.01) were significantly increased in Eco group. Compared with Con group, two families (Tannerellaceae and Xanthobacteraceae) and 4 genera (*Sphingomonas*, *Acinetobacter*, *Collinsella*, *Ralstonia*) in Eco group were significantly increased (*p* < 0.05 or *p* < 0.01). Moreover, it should be noted that the relative abundance of families (Tannerellaceae, Xanthobacteraceae, Burkholderiaceae) and genera (*Sphingomonas*, *Acinetobacter*, *Collinsella*, *Ralstonia*, *Rhodococcus*, *Herbaspirillum*) decreased significantly following FMT than Eco group (*p* < 0.05 or *p* < 0.01).

Furthermore, noteworthy variations in the microbiota among groups also were found using LEfSe analysis (LDA Score > 5) (Fig. 4E, F). Phylum Proteobacteria, family Enterobacteriaceae, and genus *Escherichia-Shigella* significantly enriched in the Eco group, whereas group FMT (phylum Firmicutes) and group Con (family Lactobacillaceae and genus *Lactobacillus*) had the different bacterial taxa enriched compared with Eco group. The changes in abundance of classification were consistent with the results in Metastats analysis. Furthermore, the phylogenetic tree at the genus taxonomic level revealed affiliations between phylum and genus levels (Fig. 4G).

### Microbial species correlation and association analysis

According to the abundance and variation of microorganisms in samples of each group, Spearman correlation analysis was performed to draw a correlation network diagram. The Top 50 relevant genera are shown in Fig. 7B. There may be covariant relationships in place that connects perturbed intestinal microbes after *E.coli* infection; however, this variant relationship may be ameliorated by FMT. Based on the correlation network analysis (Additional file 1: Table S2), the co-existence relationship of species and cooperation or antagonism patterns information among species can be identified, and the mechanism of phenotypic differences among groups could be further explained. Compared to Con group, the increases in the genera *Escherichia-Shigella* in Eco group were positively correlated with the up-regulated *Acinetobacter* and *Sphingomonas*. Another connection includes the prevalent *Lactobacillus* genera (significantly more abundant in FMT group than in Eco group) in negative associations with the *Escherichia-Shigella*, *Rhodococcus*, *Ralstonia*, *Acinetobacter*, *Sphingomonas*, and *Collinsella*. Therefore, we hypothesized that the FMT strategy modulated clinical phenotype in E. coli infected mice, suggesting a beneficial host-microbiome correlation.

## Discussion

In the current study, we demonstrated that *E.coli* induce disturbance in the intestinal microbiome that aggravate infection and damage to gut with decrease body weight and increase in mortality rate. These findings could be connected with a substantial increase in gut taxa associated with health threats. The effectiveness of FMT in treating IBD (inflammatory bowel disease) and CDI has bolstered interest in investigating its potential application. The key therapeutic role of FMT in patients might be achieved by altering gut microbiota [24]. In the current study, we observed that FMT relieved the damage to the liver and intestines. Moreover, several specific tight junction proteins (TJPs), including ZO-1, Occludin, and Claudin-1, were altered in the intestine following FMT therapy. The intact intestinal barrier could prevent the passage of invasive pathogens through the epithelium and limit intestinal inflammation [25]. Surprisingly, FMT effectively reduced a load of pathogens, increased the survival rate, and changed the structure and composition of the existing gut microbiota, which is in line with previous results [26].

The microbiome refers to the microorganisms that remarkably affects several physiological processes, including promoting the maturation of immune functions, reproductive function, even maintaining oral cavity health, etc. [27–29]. The microbiota comprises the whole microbial communities, intertwining with the host's physiological process that further forms an intestinal environment for maintaining health. However, considering its profound effects on host physiology, it is not surprising that certain shifts of microbiome compositions allow pathogens to manifest, that associated with several diseases, including IBD, cancer and obesity [30–32]. The findings of such associations provide crucial clues into host health and contribute to a series of phenotypes regarding microbial taxonomy.

Despite the recent research output in antibacterial therapy, intestinal pathogens infection remains a severe disease connected with high morbidity and mortality [33, 34]. Among these, *E. coli* is the most common bacterium (Gram-negative) and the main inducement of invasive bacterial infections [35]. The virulence factors of *E.coli*, such as lipopolysaccharide (LPS), mediate over-expression of pro-inflammatory mediators and cause systemic inflammation and damaged organs [36]. In *E. coli* infected mice, we observed decreased weight gain, increased mortality, and damaged intestinal villi. Our findings were consistent with the previous experiments that food-borne pathogens, including *E.coli* could cause serious gut pathological tissue injury and inflammation [37]. In the past decade, antibiotics have been widely used to treat bacterial diseases, while antibiotic abuse poses a serious threat to the dynamically balanced intestinal microbiome [38]. Previous research reported that a disturbed gut microbiome affects gut barrier function, that enhance the invasion into the blood circulation and infect other organs [39, 40]. Specifically, gut pathogenic bacteria can damage the intestinal barrier structure by changing the expression of TJPs, which eventually causes and creates vantage conditions for pathogens to invade and infect the body [41, 42]. Some gut infectious diseases (e.g., *Citrobacter rodentium* infection) usually involve in disruption of intestinal permeability, followed bacterial ligands into the systemic circulation through “leaky gut” induced systemic inflammation [43]. The role of TJPs in the physiology of the gastrointestinal system deserves special investigation. For example, the gastrointestinal tract epithelium does not allow many substances (e. g., pathogens) to pass through the epithelium while allowing specific substances (ions and solutes) to maintain a delicate and dynamic balance [44]. In our current study conveyed that FMT might maintain the barrier function of the epithelium to relieve the damage caused by infection. Still, further research is needed to investigate the specific mechanism.

The gut microbiome, composed of more than 1000 bacterial microorganisms that maintain gastrointestinal homeostasis, affects intestinal barrier health [6]. The gut microbiota is also a part of the gut barrier, that clears intestinal pathogens, promotes immune homeostasis, molecular nutrition, and metabolism [45]. However, several studies have demonstrated that dysbiosis might cause intestinal barrier damage, accompanied by increased intestinal permeability [46, 47]. Therefore, further investigation of the gut microbial communities was performed to reveal both taxonomic and functional characteristics in the microbiome in our current study. According to our findings, NMDS, UPGMA, and clustering heatmap analyses showed a divergence of both taxonomic and similarity in communities’ composition between Con and Eco microbiomes. However, samples in the Con and FMT groups were clustered at smaller distances, which supported the idea that the host microbiota is dynamic and variable.

Notably, the richness and diversity of species in Con group were lower than the mice challenged by *E.coli* (i.e., Eco and FMT mice). These results could have been caused by infection-associated functional redundancy, i.e., a mutual synergy between pathogenic bacteria. Further investigation should involve specific classification changes that may involve significant differences in diversity and abundance. It may help in critically ill patients, suffering disordered microbiota, characterized by loss of commensal phyla (e.g., Firmicutes), and dominant pathogens belonging to the phylum Proteobacteria [48]. In addition, it has been proposed that the changes in the proportions of Firmicutes and Bacteroidetes can predict patient outcomes as these phyla are associated with an extreme imbalance in gut microbiota [49]. In our findings, the phylum Actinobacteria was enriched in the Eco group, which has been reported to cause various infections in animals, humans, and plants [50]. The significantly enriched taxa (family Lactobacillaceae and genus *Lactobacillus*) and decreased taxa (family Enterobacteriaceae and genus *Escherichia-Shigella*) were found in Con and FMT groups than Eco group suggested the similarity of the samples between Con and FMT groups. Bacteria of the family Lactobacillaceae play an essential role in fermented foods, human health, and chemical industries [51, 52]. *Lactobacillus* is a major genus belonging to this family, which has earned extraordinary attention in the gastrointestinal tract because of its health-promoting properties [53]. While Enterobacteriaceae is a family of gram-negative which can induce various diseases in humans and animals, for example, osteomyelitis, urinary tract infections, bacteremia, etc. [54]. Significantly, among these microbial species whose relative abundance increase in *E.coli* infection, normally have two families (Tannerellaceae and Xanthobacteraceae) and four genera (*Sphingomonas*, *Acinetobacter*, *Collinsella*, and *Ralstonia*), regarded as pathogens that threaten host health. Tannerellaceae had long been recognized to be closely connected with gastrointestinal diseases [55], while *Sphingomonas* and *Acinetobacter* were found to enrich in the gut of pathogen-infected groups [56]. Oral *Collinsella* supplements reduced the expression of TJPs in enterocytes and induced “gut leakage”, which were features linked to metabolic endotoxemia [57, 58]. The *Ralstonia* genus can cause significant infection in cystic fibrosis patients, and most species that belong to this genus are regarded as pathogenicity microorganisms and cause severe disease in patients [59]. This conveyed that pathogen infection led to the reduction of beneficial bacteria through a synergistic effect or that the reduced abundance of potentially beneficial bacteria exacerbated an increase in the richness and diversity of pathogenic bacteria. Compared with Eco group, it was striking that phylum actinobacterium, family Burkholderiaceae, and genus (*Rhodococcus* and *Herbaspirillum*) were lower abundant in FMT group. These microorganisms share common characteristics: (a) the richness increase in the pathogen-infected gut by cooperative interaction, supported by results in microbial species’ correlation analysis; (b) Adapt, maintain and even promote intestinal microflora disturbance in infection [50, 60, 61]. These findings provide evidence that a beneficial host-microbiome correlation might be built under FMT therapy that provides a novel idea to relieve gut infections and pathogens-associated diseases. Moreover, the current experiment had two limitations: (1) Low resolution taxonomic identification (i.e., genus level). To address this limitation, shotgun metagenomics sequencing could be used to the species level for more accurate results. (2) The current study’s findings might not be entirely consistent with the results of clinical trials because of species differences, so clinical trials are necessary for further investigation.

## Conclusions

In summary, the current study showed the therapeutic benefits of FMT in *E.coli* infection in mice. Given these beneficial effects on intestinal disease, studies on discovering the interaction between microbiota and host to modulate the intestinal microbiome have attained significant attention. Conspicuously, we found that FMT mayreverse the disruption of intestinal villi and barrier function during infection. It suggests the therapeutic application of FMT to treat gut infections and diseases associated with functional gastric disorders. Hence, we hypothesized that a beneficial host-microbiome correlation might be built under FMT therapy, which could provide an in-depth understanding for pathogens associated with intestinal diseases.

## Supplementary Information

**Additional file 1: Table S1.** The primers information of target genes in qRT-PCR. **Table S2.** Co-existence relationship analysis among genera.

## Data Availability

The raw sequence data of current study had been submitted to the Sequence Read Archive (SRA) with accession no. PRJNA869548.
