# Supplementary material for: Fecal microbiota transplantation attenuates Escherichia coli infected outgrowth by modulating the intestinal microbiome
Source: Microb Cell Fact. 2023 Feb 17;22:30. doi: 10.1186/s12934-023-02027-z (PMC9936653; doi:10.1186/s12934-023-02027-z)
Supplement: Supplementary file 1 — Additional file 1: Table S1. The primers information of target genes in qRT-PCR. Table S2. Co-existence relationship analysis among genera. [file 12934_2023_2027_MOESM1_ESM.doc]

Table S1 The primers information of target genes in qRT-PCR.

| Primer | Forward | Reverse |
| --- | --- | --- |
| Claudin-1 | KSWGGGTTTCATCCTGGCTTCTC | YYTGAGCGGTCACGATGTTGTC |
| ZO-1 | MWYCCGAAACTGATGCTGTGGATAG | MSSCCTTGGAATGTATGTGGAGAG |
| Occludin | KKCCTTTTGAAAGTCCACCTC | RKKCAAATATGGCGATGCAC |
| ACTIN | BSCTGTCCCTGTATGCCTCTG | BSWTGTCACGCACGATTTCC |

Table S2 Co-existence relationship analysis among genera.

| Taxa | Target | Correlation |
| --- | --- | --- |
| *Lactobacillus* | *Escherichia-Shigella* | negative |
| *Lactobacillus* | *Rhodococcus* | negative |
| *Lactobacillus* | *Ralstonia* | negative |
| *Lactobacillus* | *Acinetobacter* | negative |
| *Lactobacillus* | *Sphingomonas* | negative |
| *Lactobacillus* | *Helicobacter* | negative |
| *Lactobacillus* | *Collinsella* | negative |
| *Escherichia-Shigella* | *Acinetobacter* | positive |
| *Escherichia-Shigella* | *Sphingomonas* | positive |
| *Rhodococcus* | *Ralstonia* | positive |
| *Rhodococcus* | *Herbaspirillum* | positive |
| *Rhodococcus* | *Sphingomonas* | positive |
| *Rhodococcus* | *Collinsella* | positive |
| *Ralstonia* | *Herbaspirillum* | positive |
| *Ralstonia* | *Acinetobacter* | positive |
| *Ralstonia* | *Sphingomonas* | positive |
| *Herbaspirillum* | *Sporosarcina* | negative |
| *Herbaspirillum* | *Sphingomonas* | positive |
| *Herbaspirillum* | *Erysipelatoclostridium* | positive |
| *Acinetobacter* | *Roseburia* | positive |
| *Acinetobacter* | *Sphingomonas* | positive |
| *Acinetobacter* | *Helicobacter* | positive |
| *Acinetobacter* | *Collinsella* | positive |
| *Sphingomonas* | *Collinsella* | positive |
| *Sphingomonas* | *Anaerostipes* | positive |
| *Collinsella* | *Anaerostipes* | positive |
